# Comparison of 6 % hydroxyethyl starch and 5 % albumin for volume replacement therapy in patients undergoing cystectomy (CHART): study protocol for a randomized controlled trial

**DOI:** 10.1186/s13063-015-0866-z

**Published:** 2015-08-28

**Authors:** Tobias Kammerer, Florian Klug, Michaela Schwarz, Sebastian Hilferink, Bernhard Zwissler, Vera von Dossow, Alexander Karl, Hans-Helge Müller, Markus Rehm

**Affiliations:** Department of Anesthesiology, Hospital of the University of Munich, LMU Munich, Marchioninistrasse 15, 81377 Munich, Germany; Department of Anesthesiology, Surgical Clinic Munich-Bogenhausen, Denninger Strasse 44, 81679 Munich, Germany; Department of Urology, Hospital of the University of Munich, LMU Munich, Marchioninistrasse 15, 81377 Munich, Germany; Institute of Medical Biometry and Epidemiology, Philipps University, Bunsenstrasse 3, 35037 Marburg, Germany

**Keywords:** Albumin, Colloids, Glycocalyx, Renal failure, Volume replacement therapy

## Abstract

**Background:**

The use of artificial colloids is currently controversial, especially in Central Europe Several studies demonstrated a worse outcome in intensive care unit patients with the use of hydroxyethyl starch. This recently even led to a drug warning about use of hydroxyethyl starch products in patients admitted to the intensive care unit. The data on hydroxyethyl starch in non–critically ill patients are insufficient to support perioperative use.

**Methods/Design:**

We are conducting a single-center, open-label, randomized, comparative trial with two parallel patient groups to compare human albumin 5 % (test drug) with hydroxyethyl starch 6 % 130/0.4 (comparator). The primary endpoint is cystatin C ratio, calculated as the ratio of the cystatin value at day 90 after surgery relative to the preoperative value. Secondary objectives are inter alia the evaluation of the influence of human albumin and hydroxyethyl starch on further laboratory chemical and clinical parameters, glycocalyx shedding, intensive care unit and hospital stay and acute kidney injury as defined by RIFLE criteria (risk of renal dysfunction, injury to the kidney, failure of kidney function, loss of kidney function, and end-stage kidney disease) criteria.

**Discussion:**

There is a general lack of evidence on the relative safety and effects of hydroxyethyl starch compared with human albumin for volume replacement in a perioperative setting. Previously conducted studies of surgical patients in which researchers have compared different hydroxyethyl starch products included too few patients to properly evaluate clinical important outcomes such as renal function. In the present study in a high-risk patient population undergoing a major surgical intervention, we will determine if perioperative fluid replacement with human albumin 5 % will have a long-term advantage over a third-generation hydroxyethyl starch 130/0.4 on the progression of renal dysfunction until 90 days after surgery.

**Trial registration:**

EudraCT number 2010-018343-34. Registered on 11 January 2010.

## Background

Various crystalloid and colloid solutions are available to the clinician for perioperative volume replacement therapy. Colloids are large–molecular weight (nominally MW >30,000) substances. In normal plasma, the plasma proteins are the major colloids present. Albumin solutions are available for use as colloids. Various other solutions containing artificial colloids, such as hydroxyethyl starches (HESs), are commonly used in clinical praxis.

Different preparations are indicated according to the different patient types and the type of fluid loss. For some decades there have been contradicting philosophies, in particular concerning the best choice for volume substitutes to deal with perioperative blood loss. However, fluid preloading and liberal intraoperative fluid substitution are not evidence-based procedures [1]. According to current reviews, recommended rational fluid therapy includes a combination of crystalloid and colloid solutions. An adequate replacement of fluid needs seems to have the power to improve patient outcomes and should be considered the therapy of choice to minimize perioperative fluid shifting [2].

HES solutions are used for the treatment of hypovolemia (low blood volume) when plasma volume expansion is desired. There is conflicting evidence about its relative safety, most notably regarding adverse effects of HES on kidney function.

In an animal model, the application of 10 % HES 200,000 already led to a reduction of diuresis and increases in inflammation and tubular damage [3]. These effects were more distinctive in 10 % HES 200,000 than in 6 % HES 130,000 or lactated Ringer solution.

In the Efficacy of Volume Substitution and Insulin Therapy in Severe Sepsis study, conducted by the German Competence Network Sepsis, the authors compared 10 % HES 200,000 with lactated Ringer solution as volume replacement therapy in critically ill patients. In the HES group, the incidence of acute renal failure, renal replacement therapy and need for blood transfusion were significantly increased. The authors postulated that the long storage of HES molecules in the tissue and a potential direct toxicity of the substance per se could be responsible for these negative effects [4]. Indeed, these results were seen only in patients who received more than 22 ml/kg/day.

Schabinski and colleagues compared the effect of 6 % HES 130/0.4 with 4 % gelatin on renal failure and mortality in critically ill surgical patients. They showed that a significant increase in mortality and acute renal failure occurred in the HES group if the cumulative dose of 33 ml/kg body weight was exceeded [5].

A survey of 120 Scandinavian intensive care units (ICUs) published in 2008 [6] revealed that, for most physicians, colloids are a second-choice volume substitute. Only one-third were found to use it as a primary volume substitute. HES 130/0.4 was the preferred colloid. A large part of the interviewees attested that there were no internal directives or contraindications for the use of HES at their centers. At the same time, almost all interviewees indicated that they would change their infusion practice if randomized controlled studies clearly showed negative effects on mortality and renal failure [6].

An alternative to HES is the use of human albumin (HA). The safety of HA was examined in the Saline versus Albumin Fluid Evaluation (SAFE) study, published in 2004 [7]. In that large investigation, the impact of saline 0.9 % and albumin 4 % was compared in critically ill patients. Neither in the whole group nor in one of the subgroups was a relevant difference found with regard to mortality, ICU length of stay, organ failure, respiratory support or renal replacement therapy. A post hoc analysis showed that the results were independent of the serum albumin value of the patients [8]. Indeed, in a subgroup analysis, the authors found an increase in mortality in patients with brain injury who received albumin compared with those in the saline group [9]. However, this was not a negative effect of the colloid albumin, but the result of volume loading in patients who are not hypovolemic and therefore without demand of any colloid infusion [10].

In a meta-analysis, Wilkes and Navickis investigated the safety of albumin and its influence on mortality. The authors compared 55 randomized trials in patients after major surgery, trauma, burn, hypoalbuminemia, ascites and other diseases. The researchers in the included trials compared albumin therapy with crystalloid therapy, no albumin or lower doses of albumin. Overall, the authors found no effect of albumin on mortality [11].

In an experimental study, Jacob et al. [12] investigated the different negative effects of colloids on vascular permeability. The authors perfused an isolated heart (guinea pig) with 5 % albumin, 6 % HES 130/0.4 and saline 0.9 % and observed the fluid extravasation before and after 20 minutes of ischemia. This study demonstrated a significantly lower extravasation in the albumin and HES group compared with the group that was given saline 0.9 %. After ischemia, a transient increase in vascular permeability resulted from use of HES and saline 0.9 %. In the albumin group, there was no increase in vascular leakage. This effect was independent of the intravascular colloid osmotic pressure. The authors concluded that albumin interacts with the endothelial glycocalyx, which seems to be the cause of the protective effects of albumin on the vascular barrier [12].

In an investigation on isolated heart perfusion, Jacob et al. [13] examined the effects of albumin on endothelial integrity and myocardial function after 4 h of ischemia. The authors compared Bretschneider solution with and without addition of albumin. After reperfusion, intracoronary adhesion of polymorphonuclear granulocytes, edema formation, left and right heart performance of pressure-to-volume work, and glycocalyx formation were assessed. The intracoronary adhesion of leukocytes was doubled in the Bretschneider solution group, whereas it remained at basal values after albumin addition. Addition of albumin also decreased edema and led to significantly better right ventricular function. Glycocalyx shedding was significantly lower in the albumin group than in the group without albumin addition. The authors concluded that addition of albumin improves endothelial integrity as well as heart function after 4-h ischemia, owing to protection of the glycocalyx [13].

The effects of HES 200/0.6 and HES 130/0.4 on renal function after transplant were compared in a retrospective investigation by Blasco et al. in brain-dead donors [14]. Thirty-two donors were included in every group. The appearance of delayed organ function and postsurgical creatinine was documented. Delayed organ function was found in the group treated with HES 130/0.4 compared with patients treated with HES 200/0.6. The creatinine levels after 1 month amounted to 133 μmol/L in the HES 130/0.4 group and 172 μmol/L in the HES 200/0.6 group (*p* = 0.005). After 1 year, increased creatinine was found in the HES 200/0.6 group compared with the HES 130/0.4 group (147 μmol/L versus 128 μmol/L; *p* = 0.05). The authors concluded that the use of modern third-generation HES preparations are associated with better postsurgical renal function [14].

Van der Linden et al. compared HES 130/0.4 with modified 3 % gelatin solution in 132 cardiac surgery patients with regard to perioperative blood loss and transfusion need. Both groups also received a cumulative dose of 48.9 ml/kg of colloids. Blood loss, transfusion need, laboratory parameters and hemodynamics were comparable in both groups. The authors concluded that it is safe to use 50 ml/kg of HES 130/0.4 [15].

In a systematic review, Hartog and colleagues [16] identified 56 randomized controlled trials on the use of HES 130/04 in an elective surgical setting. These studies were small-sized and of short duration. The main goal was to assess whether published studies on HES 130/0.4 resuscitation are sufficiently well designed to make conclusions about the safety of this compound. The 56 studies were small, heterogeneous, and involved different control fluids and different clinical conditions. The authors concluded that the results of these studies could not be pooled and that the studies did not provide convincing evidence that third-generation HES 130/0.4 is safe in surgical, emergency, or ICU patients, despite publication of numerous clinical studies [16].

Recent data have associated the use of HES products with an increased risk of severe adverse events (SAEs) when used in certain patient populations. After a review of the available evidence [17], on 14 June 2013, the Pharmacovigilance Risk Assessment Committee of the European Medicines Agency concluded that the benefits of HES solutions no longer outweighed their risks and recommended that the marketing authorizations for these medicines be withdrawn [18]. The European Union regulatory agency then decided to restrict the indication for HES solutions. HES is now indicated only as second-line treatment after crystalloids when there is acute bleeding. The use of HES solution is contraindicated in critically ill patients, including those with sepsis, burns, transplant and cerebral bleeding, and renal impairment and/or hepatic dysfunction. On 24 June 2013, the U.S. Food and Drug Administration recommended that HES products not be used in critically ill patients or in those with preexisting renal dysfunction, but it also did not withdraw them completely.

Despite of the fact that there are several small clinical trials in which HES was compared with another fluid for volume replacement in various clinical settings, there is a general lack of evidence on the relative safety and efficacy of HES versus HA for volume replacement in the perioperative period. Previously conducted studies of surgical patients in which different HES products were compared included too few patients for proper evaluation of important clinical outcomes, such as renal function.

In the present study, we will determine, in a patient population undergoing a major surgical intervention, if perioperative fluid replacement with HA has a long-term advantage compared with a third-generation HES (HES 130/0.4) on the progression to renal dysfunction until 3 months after surgery.

### Hydroxyethyl starch 6 % 130/0.4 (Volulyte)

Blood and protein losses that occur during surgical procedures can be compensated by the patients up to a certain point. The current infusion practice is to provide a combination of crystalloid and colloid solutions. The objective is to reach hemodynamic targets and treat the diuresis of the patient to maintain sufficient organ perfusion. Guided by the hemoglobin values, blood transfusions are also used.

Protein and blood losses are currently substituted, at least in Central Europe, with HES. The common modern preparation is 6 % HES with a MW of 130 kDa and a substitution degree of 0.4 (Volulyte; Fresenius Kabi, Bad Homburg, Germany). This solution contains, in comparison with the serum value, higher sodium and chloride amounts (154 mmol/L in each case). According to the manufacturer, the maximum dose to be administered is 50 ml of hydroxyethyl starch/kg (i.e., 3500 ml for a patient of 70-kg body weight). This dose is based on experimental investigations in rats where the infusion of 9 g/kg HES showed no toxic effects. Information concerning the maximum dose to be administered in humans is absent.

Up to now, HES preparations of the modern generation were examined only in specified patient groups. No publication of a systematic investigation with information about maximum administrable dose exists in the literature. Therefore, it is unclear whether organ-toxic or coagulation-restraining effects, as proved with older HES preparations, also occur with use of the modern products.

### Human albumin 5 % (Humanalbin)

HA is a natural component of blood plasma and is responsible for colloid osmotic pressure. It has a MW of 66 kDa and acts as a transport protein for water-indissoluble substances in the blood. It binds cations as well as anions and contributes on account of these ampholytic qualities to the buffer capacity. In addition, it is an important component of the endothelial glycocalyx, which forms the so-called endothelial surface layer, together with the endothelial cell line.

HA as a colloidal volume substitute is a content of various clinical and experimental investigations. Still, there is controversial international discussion regarding this substance as infusion solution. The SAFE study investigators examined this aspect in the most recent study. Indeed, those authors compared HA with a crystalloid solution (saline 0.9 %). The safety of this substance could be proved in the whole group, even though a subgroup showed conflicting results [9].

The HA solution used in this investigation is a 5 % infusion solution (Humanalbin; CSL Behring, Marburg, Germany) with at least 96 % HA. The substance is licensed for volume replacement therapy. Dosage and infusion rate are recommended to be adjusted to the patient’s individual requirements. There is no upper dose limit.

### Trial rationale

In the present trial, data concerning postoperative renal function are the main interest, in addition to blood loss, need for transfusions and coagulation substitutes, hospital stay, and complications and mortality. Patients undergoing cystectomy will be included. These patients are considered high-risk patients for the development of progressive renal dysfunction postoperatively based on their underlying disease and the severe surgical intervention. Because of its higher accuracy, cystatin C was chosen for calculation of glomerular filtration rate (GFR) [19, 20].

Both colloid solutions, HES and HA, are used in routine clinical perioperative settings. Their comparative safety and long-term effects on renal function in perioperative patients has not been studied in well-controlled and adequately powered randomized clinical trials. It is expected that the findings of this trial will increase knowledge about fluid management in surgical patients who are at risk for developing renal dysfunction postoperatively.

### Trial objectives and endpoints

#### Primary objective

Our primary objective is to compare the effects on renal function of HA 5 % with those of HES 130/0.4 when administered for perioperative volume replacement in patients undergoing cystectomy, with the aim of demonstrating the superiority of HA over HES.

#### Primary endpoint

The primary endpoint is the cystatin C ratio, calculated as the ratio of the cystatin value at day 90 after surgery relative to the preoperative value. This ratio corresponds to the calculated GFR ratio (the value at day 90 relative to that before surgery). The calculation of the GFR is carried out with the help of cystatin C, based on the following formula:

$$ \mathrm{G}\mathrm{F}\mathrm{R}\ {\left(\mathrm{ml}/ \min \right)}_{\mathrm{calculated}} = 74,835 \times \mathrm{cystatin}\ \mathrm{C}\ {\left(\mathrm{mg}/\mathrm{L}\right)}^{-1333} $$

#### Secondary objectives

Secondary objectives of the clinical investigation are the evaluation of the influence of HA and HES on further laboratory chemical and clinical parameters, ICU and hospital length of stay and acute kidney injury as defined by RIFLE criteria (risk of renal dysfunction, injury to the kidney, failure of kidney function, loss of kidney function, and end-stage kidney disease). Also, we are interested in the presence of pruritus evaluated by conducting a standardized interview.

#### Secondary endpoints

- Incidence of acute kidney injury as defined by RIFLE criteria (in hospital and at midterm [3 months]) (see Appendix: Table 4)
- Relative change of calculated GFR (by cystatin C) up to the third postoperative day
- Glycocalyx shedding (syndecan 1, hyaluronan) at days 0, 1 and 3
- Glomerular damage as measured by neutrophil gelatinase-associated lipocalin at days 0, 1, 3 and 90
- Length of ICU and hospital stay
- Necessity and duration of renal replacement therapy
- Presence of pruritus at day 90

#### Further objectives and variables

We also want to investigate the effect of the fluid therapy on the thrombocyte function and coagulation as measured using the Multiple Platelet Function Analyzer (Multiplate; Roche Diagnostics, Mannheim, Germany), Platelet Function Analyzer (PFA 100; Siemens Healthcare, Erlangen, Germany) and rotational thrombelastometry (ROTEM; Tem international GmbH, Munich, Germany). Also we are interested in the life quality after the operation evaluated by standardized interviews. Furthermore, we will collect the following data:

- Intraoperative blood loss, urine output and fluid amount
- The laboratory chemical course of creatinine and urea, starting before surgery up to the third postoperative day and at 90 days (visit 5)
- The laboratory chemical course of serum albumin, starting presurgically up to the third postoperative day
- Mortality
- Dementia screening (Mini Mental State Examination) on the day of screening
- Delirium screening (Nursing Delirium Screening Scale) postoperatively in the recovery room and at days 1 and 3
- Life quality assessment preoperatively and at day 90 (activities of daily living, instrumental activities of daily living)
- Effect on thrombocytes and coagulation measured by ROTEM, PFA 100) and Multiplate at day 0
- Incidence of adverse events and SAEs

## Methods/Design

In this single-center, open-label, randomized, comparative trial, we will investigate two parallel patient groups, comparing HA (test drug) versus HES (comparator). Ethical approval of this trial was obtained from the ethics committee of the Ludwig Maximilians University of Munich (reference number 311-11). The confirmatory statistical analysis is based on a leading surrogate parameter of renal function, with the aim of establishing a recommendation for therapy optimization. Because both investigational medicinal products (IMPs) are licensed for volume replacement in the perioperative setting in this trial, it is a phase IV trial. However, the clinical aim corresponds to that of a confirmatory phase IIb or III trial.

Randomization will be performed by stratifying participants by type of surgical procedure (ileum conduit or neobladder) to balance allocation to treatment groups with respect to this risk factor for postoperative renal dysfunction.

After randomization, each patient will receive the allocated volume replacement treatment according to the current prescribing information up to the seventh operative day. The patient should be kept on the allocated infusion solution during the first 90 days after operation if further volume replacement therapy becomes necessary, if not contraindicated, and if manageable.

A detailed schedule of study activities, and the treatment algorithm to be used for fluid management in both treatment arms are provided in the Appendix: Tables 1, 2 and 3.

### Anesthetic management

All patients will receive thoracic epidural anesthesia in combination with general anesthesia before surgery. Patients with contraindications to neuraxial procedures (e.g., dual platelet inhibition) will receive general anesthesia and postoperative patient-controlled analgesia with piritramide. The anesthetic technique used will be standardized. Anesthesia will be induced with propofol (2 mg/kg), sufentanil (0.4 mg/kg) and rocuronium (0.6 mg/kg) and maintained with propofol and remifentanil or sevoflurane in patients with certain conditions (e.g., cardiopulmonary diseases). After surgery, the epidural will be maintained for at least 3 days and will be combined with acetaminophen, non-steroidal anti-inflammatory drug and an opioid (piritramide) if needed. Intraoperative hemodynamic monitoring will be performed with a Vigileo monitor with FloTrac sensor (Edwards Lifesciences, Irvine, CA, USA). After randomization, patients will receive either HA or HES from one of the investigators according to an algorithm (see Appendix).

### Postoperative care

American Society of Anesthesiologists Physical Status (ASA) classification I and II patients without cardiopulmonary diseases or cerebral insufficiency will be transferred directly from the recovery room to the general ward. ASA classifications III and IV patients or those with perioperative complications will be transferred from the operating room to the ICU for at least 1 day. All patients will receive early postoperative enteral feeding without a nasogastric tube as well as early mobilization. All patients will be visited by in-hospital postoperative pain management service staff daily.

### Treatment groups

After screening and randomization, every patient is assigned to one of the two therapy arms:

1. *VoluCyst study arm* (patients with cystectomy treated with HES): In this arm, patients receive 6 % HES 130/0.6 (Volulyte) perioperatively and up to the third postoperative day as the only colloid according to a treatment algorithm (see Appendix). The maximum dose according to the prescribing information is 50 ml/kg/day (e.g., 3500 ml for a patient with a body weight of 70 kg). Patients who require additional volume replacement therapy from day 3 until discharge will be treated primarily with HES, unless a contraindication to HES has emerged.
2. *AlbuCyst study arm* (patients with cystectomy treated with HA): In this arm, patients receive 5 % HA (Humanalbin) perioperatively and up to the third postoperative day as the only colloid according to a treatment algorithm (see Appendix). Patients who require additional volume replacement therapy from day 3 until discharge will be treated with HA.

### Trial population and selection criteria

Patients will be screened for eligibility by using the surgery schedule. Patients who seem to be eligible for study participation based on their diagnosis are screened according to the inclusion and exclusion criteria. Patients who fulfill these criteria are informed about the present study.

#### Inclusion criteria

Subjects must meet all of the following inclusion criteria to be eligible for enrolment into the trial:

- Patients (male and female) aged 18–85 years
- Patients undergoing cystectomy with urinary diversion using an ileal conduit or neobladder procedure
- Ability to follow study instructions and likely to attend and complete all required visits
- Written informed consent provided

#### Exclusion criteria

Subjects presenting with any of the following exclusion criteria may not be included in the trial.

##### General exclusion criteria

- Unfavorable prognosis (e.g., palliative surgical care in cases of obstruction of the efferent urinary tract)
- Evidence of metastatic disease
- Bleeding tendency or platelet dysfunction
- Preoperative creatinine clearance <30 ml/min
- Preoperative chemotherapy with nephrotoxic drugs (e.g., cisplatin)
- Application of >1000 ml of colloid solution within the 24 h before surgical intervention
- Physical or acute medical condition, psychiatric condition or laboratory abnormality that, based on the investigator’s decision, may put the patient at risk, may confound the trial results or may interfere with the patient’s participation in this clinical trial
- History of hypersensitivity to the investigational drug or to drugs with a similar chemical structure
- History of uncontrolled chronic disease or a concurrent clinically significant illness or medical condition that, in the investigator’s opinion, would contraindicate study participation or compliance with protocol-mandated procedures
- Known or persistent abuse of medications, drugs or alcohol
- Simultaneous participation in another clinical trial or participation in any clinical trial involving an IMP within 30 days before provision of written informed consent for this trial

##### Special restrictions for women

- Current or planned pregnancy or nursing women
- Women of childbearing potential who are not using and not willing to use medically reliable methods of contraception for the entire study duration (such as oral, injectable or implantable contraceptives or intrauterine contraceptive devices), unless they are surgically sterilized and/or hysterectomized or there are any other criteria considered sufficiently reliable by the investigator in individual cases

#### Subject information and recruitment

If a patient appears to be eligible for the study, either the investigator responsible for that site or delegated medical doctors will provide the patient a full verbal explanation of the trial and the Patient Information Sheet so that the patient can consider participating. This will include detailed information about the rationale, design and personal implications of the study. After information is provided to patients, they will have sufficient time to consider participation before they are asked whether they would be willing to take part in the trial.

It is imperative that written consent be obtained before any trial-specific procedures commence. The investigator will then record the details of these trial patients in trial-specific lists.

#### Randomization and stratification

This trial is designed as an open-label trial. Randomization will be performed at the Institute for Medical Informatics, Biometry and Epidemiology (IBE) of the University of Munich, and the treating physicians will be informed about the treatment arm to which a patient is assigned.

Randomization to both treatment arms will be performed in a ratio of 1:1. The randomization technique is based on randomized, balanced blocks with random block length. The procedure considers stratification by type of surgical procedure (ileal conduit or neobladder). The IBE will provide an internet-based randomization tool (Randoulette), which chooses the colloid treatment group for a new patient fulfilling the eligibility criteria and having signed the informed consent. Randoulette will register the patient by the patient’s pseudonym or screening number, sex, year of birth and stratum (ileal conduit or neobladder) before the allocated colloid treatment group is provided.

### Statistical planning and analysis

#### Statistical analyses

The primary efficacy analysis statistically tests superiority of HA versus HES at an α significance level of 5 % (two-sided, simultaneously testing both sides at a level of 2.5 %) with respect to the primary endpoint.

The primary endpoint is expected to be non-normally distributed. After logarithm transformation, the primary endpoint may not be normally distributed. Thus, the corresponding statistical null hypothesis is the hypothesis that the distribution functions of the endpoint in the HA group and the HES group are equal and will be tested non-parametrically with the Mann-Whitney-Wilcoxon test to detect directed differences of the distributions. The Mann-Whitney-Wilcoxon test will be applied. The one-sided *p* value will be calculated, if possible, using Fisher’s exact test.

The primary endpoint adjusts in a specific way for the preoperative cystatin C values. At the planning stage, the Mann-Whitney-Wilcoxon test will be applied in the stratified version by type of surgical procedure (ileal conduit or neobladder). If adjustments of stratification are deemed necessary during the course of the trial, the trial protocol will be amended.

A fixed-sample design is planned. Changes of trial design, if necessary or advisable, resulting from an interim analysis without significance testing (no αspent) had to keep the prespecified significance level. In that case, the Brownian motion will be used for modeling the primary test statistic based on the accumulating data as a stochastic process (see next section).

The distributions will be described at all time points by median, minimum, maximum and quartiles separately for both the HES and HA groups.

The primary statistical analysis will be based on the intention-to-treat (ITT) principle.

Analysis of all secondary endpoints and of patient characteristics will be descriptive. Descriptive comparisons will be conducted with the *t* test or, in the case of deviations from the normal distribution, with the Mann-Whitney-Wilcoxon test, or with Fisher’s exact test in case of a binary outcome.

Safety data will be analyzed descriptively in the two groups at least every 12 months.

#### Interim analyses regarding confirmatory analysis

An interim analysis will be performed as approximately a midcourse analysis with the objective of checking the assumptions of the initial sample size calculation. There is no α spending prespecified at the planned interim analysis. In cases where the interim analysis results in substantial differences from the assumptions at the planning stage, the assumptions will be revised and a sample size recalculation will be performed. The sample size recalculation will be based on the conditional rejection probability approach (see next section). If a sample size modification deems advisable, the study protocol will be amended.

Calculations for sequential analyses regarding the primary confirmatory analysis will be done in the Brownian motion model. Values of a Brownian motion are approximately resembled based on the accumulating data and applying the inverse normal transformation of the (exact) one-sided *p* values at interim and final analyses followed by multiplication with the square root of the information time. The information time is approximated by

$$ \frac{{\mathrm{n}}_{\mathrm{IC},\mathrm{H}\mathrm{A},\mathrm{I}}\cdot {\mathrm{n}}_{\mathrm{IC},\mathrm{H}\mathrm{E}\mathrm{S},\mathrm{I}}}{{\mathrm{n}}_{\mathrm{IC},\mathrm{H}\mathrm{A},\mathrm{I}}+{\mathrm{n}}_{\mathrm{IC},\mathrm{H}\mathrm{E}\mathrm{S},\mathrm{I}}+1}+\frac{{\mathrm{n}}_{\mathrm{NB},\mathrm{H}\mathrm{A},\mathrm{I}}\cdot {\mathrm{n}}_{\mathrm{NB},\mathrm{H}\mathrm{E}\mathrm{S},\mathrm{I}}}{{\mathrm{n}}_{\mathrm{NB},\mathrm{H}\mathrm{A},\mathrm{I}}+{\mathrm{n}}_{\mathrm{NB},\mathrm{H}\mathrm{E}\mathrm{S},\mathrm{I}}+1} $$

where n_S,T,I_ denotes the number of patients with assessed primary endpoint in stratum S (S = IC for ileal conduit or S = NB for neobladder) and treatment group T (T = HA or T = HES) at an arbitrary time during the course of the trial. Alternatively, a proportional information time scale may be used (e.g., by multiplication by a factor of 4).

#### Modifications of the statistical design for confirmatory analysis

To keep the type I error level in the case of a design modification at any time during the course of the trial, the Conditional Rejection Probability approach of Müller and Schäfer will be applied [21, 22]. Thereby, as described in the preceding section, the calculations will be based on the Brownian motion approximation. In the case of a modification of the statistical design (e.g., a sample size recalculation), the trial protocol will be amended.

#### Power considerations and sample size calculation

Three parameters will influence the sample size of the study, in which we will use the Mann-Whitney-Wilcoxon test for the statistical decision based on a fixed-sample design: the level of significance, the power of the two-sided test and the probability that an observation X_AlbuCyst_ in the HA group is less than an observation X_VoluCyst_ in the HES group. For sample size planning, no stratification and approximately normally distributed data for log-transformed cystatin C values and calculated log-transformed GFR values are assumed. The location parameter and standard deviation of cystatin C at baseline are assumed to be 0.9 mg/L and 0.2 mg/L, respectively, based on the publication by Evangelopoulos and colleagues [23]. Thereby we consider the older patient groups and greater variability because subgroups are pooled and study patients did not represent a healthy population.

For sample size calculation, the value of 0.2222 for the standard deviation of the difference at day 90 minus baseline of the log-normal transformed cystatin C values is specified in both treatment groups. There are two arguments for this specification. First, the value is suggested to be conservative in the four groups (two treatments times two measurement time points) considered in the statistical analyses. Second, adjusting for baseline values by using cystatin C values at day 90 relative to the same patients’ value at baseline is suggested to reduce rather than to increase the standard deviation.

An increase of the GFR by the factor 1.2 due to HA compared with HES would be clinically meaningful and worthwhile to be detected as statistically significant difference at a two-sided type I error level of α = 5 % with a statistical power of 1 − β = 80 %. For the interpretation of the increase of the GFR by the factor 1.2 consider, for example, a patient with a Cystatin C value of 0.9 mg/L. This value results in a calculated GFR at baseline between 86 and 87 ml/min. Then, the factor 1.2 would mean an increase of GFR at day 90 from 60 to 72 ml/min, from 65 to 78 ml/min, or from 70 to 84 ml/min.

The factor of 1.2 with respect to GFR transfers to the detectable difference of 0.1368 for the log-transformed (natural logarithm) Cystatin C values when using the conservative formula where the exponent is −1.333.

The detectable difference and the specified standard deviation correspond to the probability of

$$ \mathrm{P}\left({\mathrm{X}}_{\mathrm{AlbuCyst}}<{\mathrm{X}}_{\mathrm{VoluCyst}}\right)=0.6683. $$

With a sample size of 47 in each group, the non-stratified Mann-Whitney-Wilcoxon test at a 0.05 two-sided significance level will have 80 % power to detect a probability of 0.6683 that an observation X_AlbuCyst_ is less than an observation X_VoluCyst_. This means that n = 94 assessed patients are required in total to achieve the desired power.

On the basis of our experience with patient compliance in previous studies and routine treatment, we expect a dropout rate of about 10 %. Thus, adjusting for slight random imbalances in allocation to the treatment groups, a total of 105 patients (50–55 in each treatment group) have to be enrolled.

It has to be taken into consideration that about 50 % of patients fulfilling the inclusion criteria for this trial might refuse to give their informed consent to participate; we therefore expect to screen a total of about 210 patients for eligibility.

#### Sensitivity analyses

After the primary ITT analysis, sensitivity analyses will follow per protocol or according to as-treated principles. In addition to the primary Mann-Whitney-Wilcoxon analysis, the Mann-Whitney-Wilcoxon test will be applied to the cystatin C values at day 90, and, assuming approximately normal distributions after log transformation (natural logarithm), explorative regression analyses will be performed modeling the log-transformed (natural logarithm) cystatin C values depending on treatment group, log-transformed (natural logarithm) baseline cystatin C values and other potentially prognostic factors.

### Ethics and Good Clinical Practice

The trial will be conducted in accordance with the International Conference on Harmonization guidance regarding Good Clinical Practice, the relevant national regulations and the Declaration of Helsinki. The study protocol and consent forms were approved by the institutional review board of the Ludwig Maximilians University of Munich (reference number 311-11).

## Discussion

Despite the fact that there are several smaller clinical trials comparing HES to another fluid for volume replacement in various clinical settings, there is a general lack of evidence on the relative safety and effects of HES versus HA for volume replacement in a perioperative setting. Previously conducted studies of surgical patients in which researchers compared different HES products included too few patients for proper evaluation of clinically important outcomes such as renal function.

The present study will determine, in a high-risk patient population undergoing major surgical intervention, whether perioperative fluid replacement with HA has a long-term advantage over a third-generation HES (HES 130/0.4) on the progression to renal dysfunction until 3 months after surgery.

## Trial status

The study was initiated as planned in May 2012. The study is expected to be completed in February 2016.

## Appendix

**Table 1:** Flowchart

Schedule of activities	Visit 1	Visit 2	Visit 3	Visit 4	Visit 5
	Screening day (day −1)	Day 0 (day of surgery)	Day 1	Day 3	Day 90
Informed consent	X				
Inclusion and exclusion criteria	X				
Demographic data	X				
Medical history (primary diagnosis)	X				
Pregnancy test for women of childbearing potential	X				
Mini Mental State Examination	X				
Life Quality Assessment (ADL, IADL)	X				X
Concomitant medications (diuretics)	X	X	X	X	X
Cystatin C, GFR, serum creatinine	X	X	X	X	X
ROTEM, Multiplate, PFA 100		X			
Randomization		X			
NGAL, syndecan 1, hyaluronan		X	X	X	
Study drug		X	X	X	
Blood products		X	X	X	
IV fluid amount		X	X	X	
Blood losses and urine output		X	X	X	
Nursing Delirium Screening Scale		X	X	X	
AEs and SAEs		X	X	X	X
Pruritus assessment					X

*ADL* activities of daily living, *AE* adverse event, *IADL* instrumental activities of daily living, *IV* intravenous, *NGAL* neutrophil gelatinase-associated lipocalin, *PFA 100* Platelet Function Analyzer 100, *ROTEM* rotational thrombelastometry, *SAE* serious adverse event2

**Table 2:** Algorithm of infusions and transfusion in the operating room and intensive care unit

Hemodynamics	
ASA I and II patients without cardiac diseases or cerebral insufficiency:	
Stroke volume variation (SVV) <12 %	
Cardiac index (CI) > 2.5 L/min/m^2^	
MAD >60 mmHg	
ASA III and IV patients or patients with cardiac diseases or cerebral insufficiency:	
SVV <12 %	
CI >2.5 L/min/m^2^	
MAD >70 mmHg	
Central venous oxygen saturation >70 % or mixed venous oxygen saturation >65 %	
To reach the desired parameter:	
Start with infusion protocol (see below)	
If not successful within 15 minutes:	
Start with norepinephrine as first-choice vasopressor	
Infusion protocol	
Replacement of urine output with Ringer’s acetate solution in a 1:1 ratio	
Additionally, 500 ml of crystalloids for insensible sweating	
Replacement of blood and protein losses dependent on treatment assignment, with either HA 5 % or HES 6 % in a 1:1 ratio up to a transfusion trigger point or a maximum of 50 ml/kg/day	
Additionally up to 1500 ml of colloids for the protein loss into the third compartment

**Table 3:** Transfusion protocol (in support of [24])

Red blood cells			
Hb (g/dl)	Risk factors	Transfusion	Evidence level
<6 g/dl	–	Yes	1c+
>6–8 g/dl	Adequate compensation, no risk factors	No	1c+
	Limited compensation or risk factors (cardiac diseases, cerebrovascular insufficiency)	Yes	1c+
	Signs of anemic hypoxia (tachycardia, hypotension, lactic acidosis, ECG change)	Yes	1c+
8–10 g/dl	Signs of anemic hypoxia (tachycardia, hypotension, lactic acidosis, ECG change)	Yes	2c
>10 g/dl	–	No	1a

*ADL* activities of daily living, *AE* adverse event, *ECG* electrocardiographic, *Hb* hemoglobin, *IADL* instrumental activities of daily living, *SAE* serious adverse event

**Table 4:** RIFLE criteria as defined by Bellomo et al. [25]

Category	GFR criteria	Urine Output (UO) criteria
Risk	Increased creatinine × 1.5 or GFR decrease > 25 %	UO <0.5 ml/kg/h for 6 h
Injury	Increased creatinine × 2 or GFR decrease > 50 %	UO <0.5 ml/kg/h for 12 h
Failure	Increase creatinine × 3 or GFR decrease > 75 %	UO <0.3 ml/kg/h for 24 h or anuria for 12 h
Loss	Persistent ARF = complete loss of kidney function >4 wk	
ESKD	End-stage kidney disease (>3 mo)	

*ARF* acute renal failure, *ESKD* end-stage kidney disease, *GFR* glomerular filtration rate, *RIFLE* risk of renal dysfunction, injury to the kidney, failure of kidney function, loss of kidney function, and end-stage kidney disease, *UO* urine output

## Abbreviations

ADL: Activities of daily living
AE: Adverse event
ARF: Acute renal failure
ARF: Acute renal failure
ASA: American Society of Anesthesiologists Performance Status classification system
CI: Cardiac index
ECG: Electrocardiographic
ESKD: End-stage kidney disease
GFR: Glomerular filtration rate
HA: Human albumin
Hb: Hemoglobin
HES: Hydroxyethyl starch
IADL: Instrumental activities of daily living
IBE: Institute for Medical Informatics, Biometry and Epidemiology
IMP: Investigational medicinal product
ICU: Intensive care unit
ITT: Intention to treat
IV: Intravenous
i.a.: Inter alia
MW: Molecular weight
OR: Operating room
NGAL: Neutrophil gelatinase-associated lipocalin
OR: Operating room
PFA 100: Platelet Function Analyzer 100
RIFLE: Risk of renal dysfunction, injury to the kidney, failure of kidney function, loss of kidney function, and end-stage kidney disease
ROTEM: Rotational thrombelastometry
SAE: Serious adverse event
SAFE: Saline versus Albumin Fluid Evaluation study
SVV: Stroke volume variation
UO: Urine output
